# Comparison of Volatile Compounds in Jingshan Green Tea Scented with Different Flowers Using GC-IMS and GC-MS Analyses

**DOI:** 10.3390/foods13172653

**Published:** 2024-08-23

**Authors:** Zhiwei Hou, Ziyue Chen, Le Li, Hongping Chen, Huiyuan Zhang, Sitong Liu, Ran Zhang, Qiyue Song, Yuxuan Chen, Zhucheng Su, Liying Xu

**Affiliations:** 1 College of Tea Science and Tea Culture, Zhejiang Agriculture and Forestry University, 666 Wusu Street, Hangzhou 311300, China; chenziyuetea@163.com (Z.C.); liletea@163.com (L.L.); zhanghuiyuan6@126.com (H.Z.); zhangran@163.com (R.Z.); songqiyue7@163.com (Q.S.); chenxm0406@163.com (Y.C.); zhuchengsu@zafu.edu.cn (Z.S.); 2 Tea Research Institute, Chinese Academy of Agricultural Sciences, Hangzhou 310008, China; 3 Hangzhou Tea Research Institute, CHINA COOP, Hangzhou 310016, China; sytoneliu@163.com; 4 Wuhu Institute of Technology, Wuhu 241006, China; xuly@whit.edu.cn

**Keywords:** scented tea, Jingshan green tea, volatile compounds, GC–IMS, GC–MS

## Abstract

Scented green tea (*Camellia sinensis*) is a type of reprocessed green tea produced by scenting with flowers. To investigate the differences in the volatiles of scented green tea processed with four different flowers (*Jasminum sambac*, *Osmanthus fragrans*, *Michelia alba*, and *Rosa rugosa*), gas chromatography–ion mobility spectrometry (GC–IMS) and gas chromatography–mass spectrometry (GC–MS) were employed to detect and identify the volatile compounds in the four types of scented teas. GC–IMS and GC–MS identified 108 and 101 volatile compounds, respectively. The key characteristic volatile compounds, namely indole, linalool, *β*-myrcene, benzyl acetate, and ethyl benzoate (jasmine tea); cedrol, (*E*)-*β*-ionone, *γ*-decalactone, and dihydro-*β*-ionol (osmanthus tea); geraniol, phenylethyl alcohol, jasmone, methyl jasmonate, hexadecanoic acid, 4-ethyl-benzaldehyde, 2-methylbutyl hexanoate, and indole (michelia tea); and 3,5-dimethoxytoluene, (*E*)-*β*-ionone, and 2-methylbutyl hexanoate (rose tea), were identified through chemometric analysis combined with relative odor activity values (ROAVs) and sensory evaluation. This study provides new insights into the formation of aroma molecular fingerprints during green tea scenting with flowers, providing theoretical guidance for infusing distinct aroma characteristics into green tea during scented tea processing.

## 1. Introduction

Tea, made from the leaves of the plant Camellia sinensis, is considered one of the three major beverages in the world and has a variety of health benefits [1,2,3]. Its rich flavor and aroma have long been favored by consumers, with the aroma in particular considered an important indicator of tea quality [4]. The production of scented tea is becoming increasingly popular due to the enrichment of the tea’s aroma. The processing of scented tea capitalizes on the olfactory emissions of fresh flowers and the absorptive properties of tea leaves, which possess a loose and porous tissue structure, enabling the efficient adsorption of the aromatic compounds released by the flowers [5]. The fragrant flowers used to make scented tea include *Jasminum sambac*, *Osmanthus fragrans*, *Michelia alba*, *Rosa rugosa*, and other varieties. Jasmine tea is a well-known traditional scented tea. According to statistics from the China Tea Circulation Association (https://www.ctma.com.cn/index/index/zybg/id/20/) (accessed on 1 July 2024), the production of jasmine tea in China reached a global sales volume of 112,500 tons and a total output value of CNY 26.76 billion in 2020. The raw materials for scented tea typically include roasted green tea, with Jingshan green tea serving as a representative type of roasted green tea. It is processed via fixation, shaping, rolling, and drying.

Scented green tea is made by using green tea as a raw material that absorbs the fragrance of flowers. The production process of scented tea includes preparing fresh flowers and raw tea, scenting the tea with the flowers, and piling and packing the tea uniformly [5]. The density, persistence, purity, and coordination of the floral fragrance with the tea aroma are important factors that influence the quality of scented tea. These olfactory attributes are regulated by volatile compounds.

The impact of the fragrance enhancement process on the volatile compounds created in the production of jasmine tea was investigated through GC–MS analysis [6]. The results indicated that an increase in the number of fragrance enhancement cycles gradually improved the aroma quality of the jasmine tea. (*Z*)-3-hexen-1-ol acetate, (*E*)-2-hexenal, 2-nonenal, (*Z*)-3-hexen-1-ol, (*Z*)-nonen-1-ol, *β*-ionone, and benzyl acetate have been identified as key aroma compounds that contribute to the characteristic aroma of jasmine tea [6]. The aroma of osmanthus black tea was analyzed using solid-phase microextraction (SPME) combined with GC–MS [7]. The results revealed that *β*-ionone, dihydro-*β*-ionone, benzeneacetaldehyde, citral, geraniol, and linalool were the characteristic aroma compounds of osmanthus black tea [7]. Scented teas, which have absorbed the aroma compounds of flowers, also exhibit varying fragrance profiles. The processing of scented tea effectively increases the diversity of tea varieties. By utilizing flowers with distinct aromatic characteristics, a single type of tea can be transformed into multiple flavored scented teas. However, there is currently a lack of research on the differences in volatile compounds across various scented teas.

The two detection technologies used in this study are commonly employed in the research of tea and other food products. Gas chromatography–ion mobility spectrometry (GC–IMS) is simple and the equipment is relatively small, providing convenience for research, including food flavor analysis [8]. It is useful for analyzing trace volatile compounds, classifying and detecting adulteration in food, and evaluating food freshness [9,10,11]. Stir bar sorptive extraction (SBSE) is characterized by its excellent reproducibility and low detection limits; consequently, it is widely employed for aroma extraction in various types of samples due to its outstanding extraction efficiency for volatile and semi-volatile compounds [12]. The stir bar sorptive extraction–gas chromatography–mass spectrometry (SBSE–GC–MS) technique is commonly employed for the identification of key aromatic compounds in tea volatiles [13].

This study employed the HS–GC–IMS and SBSE–GC–MS techniques to identify volatile compounds in scented teas and the raw material green tea and to establish aroma fingerprint profiles for different types of scented teas. Additionally, chemometrics and the relative aroma activity value (ROAV) were used to analyze the characteristic aroma compounds of various scented teas. The results reflected the diverse impacts of different flower types on the aromatic profile of reprocessed Jingshan green tea, revealing the distinctive aroma components associated with four types of scented teas. These results established a theoretical foundation for the scenting process used to transform green tea into scented tea.

## 2. Materials and Methods

### 2.1. Sample Preparation and Collection

The fresh tea leaves were harvested in March 2023 from tea gardens in Yuhang District, Hangzhou City, Zhejiang Province, following the standard process of collecting one bud and two leaves. Subsequently, the Jingshan green tea samples were prepared via fixation, shaping, rolling, and drying. Four kinds of fresh flowers, including *Jasminum sambac*, *Osmanthus fragrans*, *Michelia alba*, and *Rosa rugosa*, were used to scent the Jingshan green tea in this study. Green tea and fresh flowers were mixed in a 1:1 weight ratio. After mixing, each sample was arranged in layers of 20 cm in height. Subsequently, the mixture was kept at 25 °C and allowed to scent for 12 h. The mixture was stirred every 3 h to dissipate the heat. After the scenting process, we separated the flowers and green tea by sieving, retaining only the green tea. The separated green tea sample was dried at 70 °C. The samples were processed in triplicate. The green tea (GT), jasmine tea (JT), osmanthus tea (OT), michelia tea (MT), and rose tea (RT) were sealed and stored at −80 °C, followed by freeze-drying before detection.

### 2.2. Chemicals and Reagents

The n-alkanes (C_6_–C_40_) and n-ketones (C_4_–C_9_) were purchased from Shanghai Aladdin Bio-Chem Technology Co., Ltd. (Shanghai, China); ethanol and sodium chloride from Sinopharm Chemical Reagent Co., Ltd. (Shanghai, China); and deionized water from Wahaha Group Co., Ltd. in Hangzhou (China). Decanoic acid ethyl ester was purchased from Shanghai Yuanye Biological Technology Co., Ltd. (Shanghai, China).

### 2.3. Volatile Compounds Detected via HS–GC–IMS

The HS–GC–IMS analysis method is an approach we have used previously [14]. In brief, a fully ground 1.0 g sample was placed in a 20 mL headspace vial. The headspace vial was placed in a headspace apparatus and incubated at 50 °C and 500 rpm for 20 min. At an operating temperature of 85 °C, the syringe needle injected 500 μL of headspace gas into the injection port through the autosampler without splitting. Subsequently, the volatile compounds were determined via gas chromatography–ion mobility spectrometry (GC–IMS) using FlavourSpec^®^. Using the MXT-5 capillary chromatography column (30 m × 0.53 mm × 1 μm, Restek Corporation, Centre County, PA, USA), the column temperature was maintained at 60 °C. GC was performed with high-purity nitrogen gas (purity ≥ 99.999%) as the carrier gas. The initial flow rate was 2.0 mL/min and was maintained at 2 min, after which it was linearly increased to 10.0 mL/min over the next 8 min. Subsequently, it increased linearly to 100.0 mL/min within 10 min. The chromatography had a runtime of 20 min. The injection port temperature was maintained at 80 °C. The ionization utilized a tritium source (^3^H); the length of the migration tube was 53 mm; the electric field strength was set to 500 V/cm; the temperature of the migration tube was maintained at 45 °C; high-purity nitrogen gas (purity ≥ 99.999%) was used as the drift gas with a flow rate of 150 mL/min; and the positive ion mode was selected for detection.

The retention time (RT) and retention index (RI) of the target compounds were established based on the calibration curve of a mixed standard containing six n-ketones (C_4_ ~ C_9_). Subsequently, the retention index of the target substances was calculated using their corresponding retention time. The qualitative analysis of the target substance was conducted by retrieving and comparing it with both the GC retention index (NIST 2020) database and the IMS migration time database using the integrated VOCal software version 6.4.

### 2.4. Volatile Compounds Detected via SBSE–GC–MS

The SBSE–GC–MS analysis method is an approach we have used previously [14]. Ground tea powder (3.0 g) was weighed into a 150 mL conical flask and brewed with 60 mL of boiled water. After steeping for 5 min, we promptly filtered the tea infusion and cooled it with chilled water. Subsequently, the tea infusion (10 mL), sodium chloride (3.0 g), and 4 μL of decanoic acid ethyl ester with a concentration of 10 mg/L were introduced into the 20 mL headspace vial. The polydimethylsiloxane (PDMS) twister (Gerstel, Muelheim, North Rhine-Westphalia, Germany) was immersed into the tea infusion, following which the volatile compounds were extracted for 90 min at 50 °C with the water bath magnetic stirrer. Finally, the twister was washed, dried, and transferred to a thermal desorption tube for subsequent GC–MS analysis.

A gas chromatograph 7890B equipped with a 5977B mass spectrometer (Agilent Technologies, Palo Alto, CA, USA) was used. GC was performed with an HP-5MS capillary chromatography column (30 m × 0.25 mm × 0.25 μm, Agilent Technologies, Palo Alto, CA, USA). The initial temperature was set at 40 °C and maintained for 5 min, followed by a ramping rate of 3 °C/min until 100 °C was reached. Subsequently, the temperature was further increased at a rate of 2 °C/min to reach 130 °C. Finally, the temperature was raised rapidly at a rate of 10 °C/min to achieve a maximum of 250 °C and held for an additional duration of 5 min. The carrier gas employed in this experiment was helium with a purity level of 99.99%. The mass spectrometer was operated in electron ionization (EI) mode at 70 eV. The temperatures of the ionization source and transmission line were 230 °C and 250 °C, respectively. Mass spectrometry employed the positive ion mode for data acquisition, encompassing a mass scanning range of *m*/*z* 30 to 350.

The thermal desorption unit (TDU, Gerstel, Germany) was operated at an initial temperature of 30 °C for 1 min. It was then ramped up to 240 °C at a rate of 100 °C per minute and held for 5 min in the splitless mode. The cooling injection unit (CIS, Gerstel, Germany) received the supply of liquid nitrogen. The liquid nitrogen was cooled to −100 °C and equilibrated for 1 min before being ramped up to 280 °C at a rate of 12 °C per second and held for 3 min.

The RT values of normal alkanes (C_6_–C_40_) were utilized to calculate the RI for all volatile compounds detected by means of GC–MS. These RI values were subsequently compared with those present in the NIST 2017 standard spectrum library and public databases (https://webbook.nist.gov/chemistry/) (accessed on 10 June 2024), enabling a qualitative analysis of the detected compounds.

### 2.5. Calculation of ROAV

The relative odor activity value (ROAV) could be calculated using the following formula: *OAV_i_* = *C_i_* / *OT_i_*, *ROAV_i_* = *OAV_i_* × (*OT_max_* / *C_max_*) × 100, where *C_i_* represents the relative content of volatile compounds, *OT_i_* denotes the odor threshold of volatile compounds, *OT_max_* corresponds to the highest odor threshold among all volatile compounds with OAV values, and *C_max_* indicates the relative content of the volatile compound with the highest OAV value [15,16].

### 2.6. Statistical Analysis

The Reporter and Gallery Plot plugins within the VOCal data processing software were utilized to generate three-dimensional spectra, two-dimensional spectra, differential spectra, and fingerprint chromatograms of the volatile compounds detected via GC–IMS. Variance analysis using the experimental data was conducted with SPSS 26.0, and principal component analysis (PCA) and orthogonal partial least squares discriminant analysis (OPLS-DA) were performed using SIMCA 14.1. TBtools and Origin 2022 were employed for visualization purposes.

## 3. Results and Discussion

### 3.1. Profiles of Volatile Compounds of Different Tea Samples

#### 3.1.1. Identification of Volatile Compounds via HS–GC–IMS

**Figure 1:**
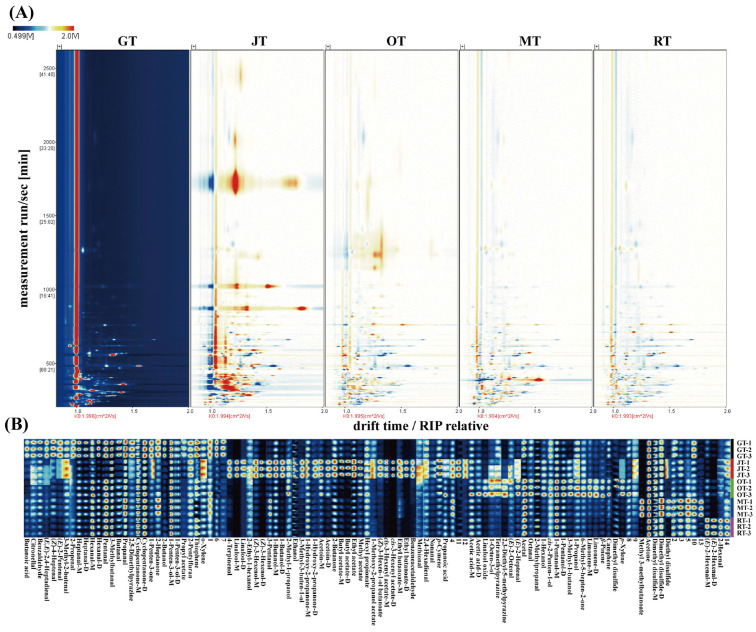
(**A**) Two-dimensional (comparative difference) fingerprint spectrum; (**B**) four types of scented teas and green tea, as determined by means of GC–IMS.

To elucidate the compositional differences in volatile compounds among the four types of scented tea and the base green tea, we employed GC–IMS to profile the volatile components of the tea samples. Tables S1 and S2 illustrate the identification of a total of 108 volatile compounds across the five samples, which were predominantly composed of alcohols, aldehydes, ketones, esters, and heterocyclic compounds. As illustrated in Figure 1B, for a more intuitive comparison of the differences in volatile compounds within the samples, the chromatogram of GT was chosen as a reference. The chromatograms of the other samples were then subtracted from this reference to generate differential comparison charts for each sample. In instances where the concentration of volatile compounds was equivalent between the target sample and the control, the resulting background subtraction had a white appearance. Conversely, red indicated that the concentration of the substance in the target sample was higher than that in the control, while blue signified a lower concentration in the target sample relative to that in the control. The majority of detection signals had retention times between 100 and 800 s, with drift times ranging from 1.0 to 2.0 s (relative RIP).

Figure 1B presents the profiles of the volatile compound fingerprints for the four types of scented tea and Jingshan green tea. Here, each sample’s distinct signal peaks are delineated in individual rows, with corresponding columns representing the identical volatile compound across various samples. The intensity of a color is directly proportional to the concentration of its components, with brighter colors indicating higher compound concentrations. The comparative analysis results indicated that there were variations in the volatile compounds of different tea samples. In the green tea (GT) samples, a higher concentration of volatile compounds such as butanoic acid, citronellal, (*E*)-2-pentenal, (*E*,*E*)-2,4-heptadienal, 2,5-dimethylpyrazine, (*Z*)-4-heptenal, benzaldehyde, 2-propenal, hexanal, 2-butanol, 3-methyl-2-butenal, and butanal were identified. In jasmine tea (JT), the volatile compounds with higher concentrations included linalool, ethyl acetate, acetic acid, (*Z*)-3-hexenyl acetate, 2-butanone, 4-terpinenol, benzeneacetaldehyde, 1-butanol, butyl acetate, methyl acetate, 1-hydroxy-2-propanone, and ethyl butanoate. Dimethyl sulfide, 1-pentanol, 2,3-diethyl-5-methylpyrazine, linalool oxide, 3-methyl-1-butanol, camphene, *β*-pinene, and limonene were present in relatively high concentrations in osmanthus tea (OT). Michelia tea (MT) exhibited a substantial composition of volatile compounds, including methyl 3-methylbutanoate, 3-pentanol, dimethyl disulfide, and 2-heptanone. Volatile compounds such as (*E*)-2-hexenal, thiophene, and 2-hexenal had high concentrations in rose tea (RT). GC–IMS analysis found pronounced discrepancies in the volatile components among the various tea samples, as evidenced by their distinct fingerprint patterns.

#### 3.1.2. Identification of Volatile Compounds via SBSE–GC–MS

**Figure 2:**
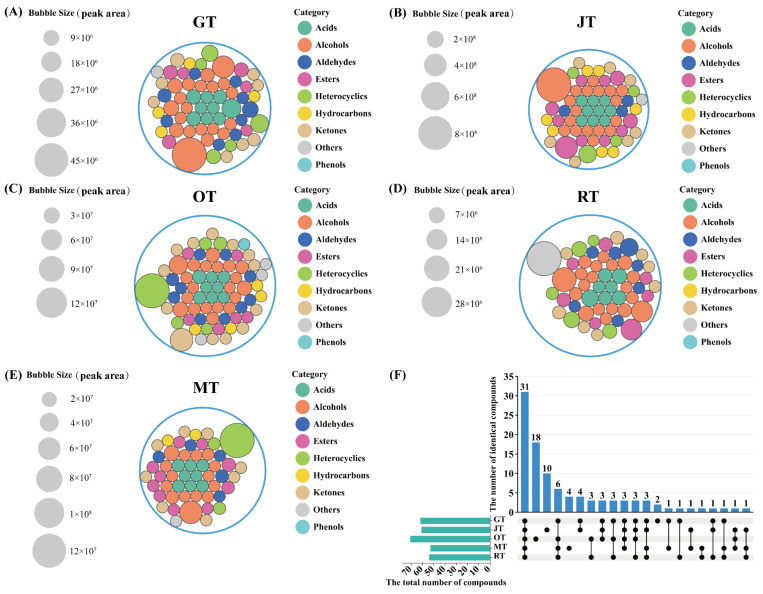
(**A**–**E**) Circle packing plots (bubble size indicates the peak area) and (**F**) UpSet diagram of the volatile profiles detected by means of GC–MS. (GT: green tea, JT: jasmine tea, OT: osmanthus tea, MT: michelia tea, RT: rose tea).

Table S2 illustrates the detection and characterization of 101 volatile compounds using GC–MS analysis. Analysis of the tea samples from GT, JT, OT, MT, and RT revealed the presence of 62, 61, 71, 53, and 54 volatile compounds, respectively, as depicted in Figure 2. GC–MS analysis identified a diverse array of compounds, consisting predominantly of 10 acids, 27 alcohols, 11 aldehydes, 15 esters, 17 ketones, 8 heterocyclics, 8 hydrocarbons, 1 phenol, and 4 others. As shown in Figure 2F, 31 compounds were found to be common to both the scented teas and the base green tea. Osmanthus tea contained 18 unique compounds, while jasmine tea contained 10 unique compounds. As illustrated in Figure 2A and Table S2, the analysis of volatile components in Jingshan green tea revealed that geraniol was present in the highest relative concentration. In jasmine tea, the relative concentration of linalool was the highest (Figure 2B). In michelia tea (Figure 2E), indole was identified as the compound with the highest relative concentration. The highest relative concentration of *γ*-decalactone was found in osmanthus tea (Figure 2C). The highest relative concentration found in rose tea was observed for 3,5-dimethoxytoluene (Figure 2D). 3,5-Dimethoxytoluene is currently acknowledged as a predominant aroma compound in several rose varieties [17] and is synthesized from orcinol through the methylation of the hydroxyl groups at two distinct positions [18].

**Figure 3:**
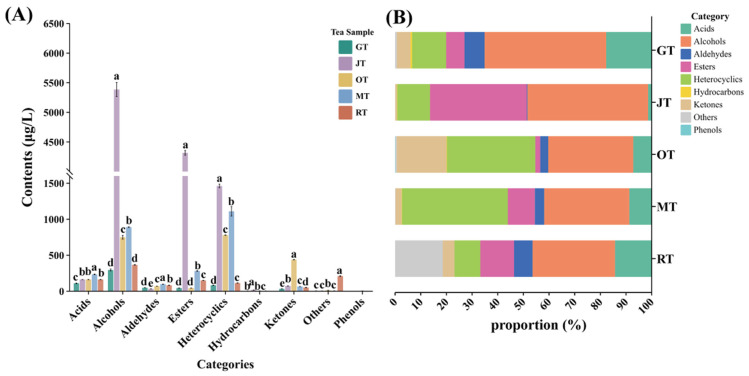
(**A**) Content of different types of volatile compounds in different tea samples (mean ± SD). The different letters on the bar chart indicate that the relative concentration of compounds is significantly different (*p* < 0.05). (**B**) Proportion of different classes of volatile compounds in different tea samples. (GT: green tea, JT: jasmine tea, OT: osmanthus tea, MT: michelia tea, RT: rose tea).

Figure 3 illustrates that the composition of alcohols and heterocyclic compounds constituted a significant proportion of the total volatile components identified in the four types of scented tea and Jingshan green tea. In different tea samples, the total amount of alcohol substances comprised 47.4% (GT), 47.0% (JT), 33.1% (OT), 33.2% (MT), and 32.1% (RT) of the total volatile compounds in each sample. The alcohols with higher concentrations included linalool and its oxides, geraniol, and phenylethyl alcohol. These volatile compounds are not only important components of tea aroma but also significant floral fragrance substances [19]. The content of heterocyclic substances in different tea samples represented 13.3% (GT), 12.8% (JT), 34.4% (OT), 41.3% (MT), and 10.1% (RT) of the total volatile compounds in their respective samples. Indole constitutes a key aroma compound within jasmine flowers, arising from the catabolism of nitrogen-containing compounds post-harvest [20]. It has been found to influence the aroma quality of jasmine tea [21]. Furthermore, in our study, we revealed a high indole content in MT, which was attributed to the adsorption of aroma compounds from *Michelia alba* by the green tea. OT contained a high concentration of heterocyclic compounds, including a notably high level of *γ*-decalactone, which distinguished it from JT and MT. *γ*-Decalactone serves as a key aroma compound in OT, delivering a profile of sweetness and coconut-like aromas [22]. Additionally, the concentration of acidic compounds was comparatively elevated in the tea samples. In JT, esters contributed a significant proportion, constituting 37.7% of the total volatile compounds present. Among the ester aroma compounds in JT, benzyl acetate had the highest content and is considered one of the characteristic aroma compounds of jasmine flowers [23]. The concentration of ketones in OT was substantially elevated when compared with that in the other tea samples. There were five ketones that were unique to OT, with particularly high levels of (*E*)-*β*-ionone. (*E*)-*β*-ionone, a compound characterized by its flowery aroma, significantly contributes to the enhancement of osmanthus tea’s scent profile [22].

### 3.2. Comparison of Volatile Compounds among Different Tea Samples

**Figure 4:**
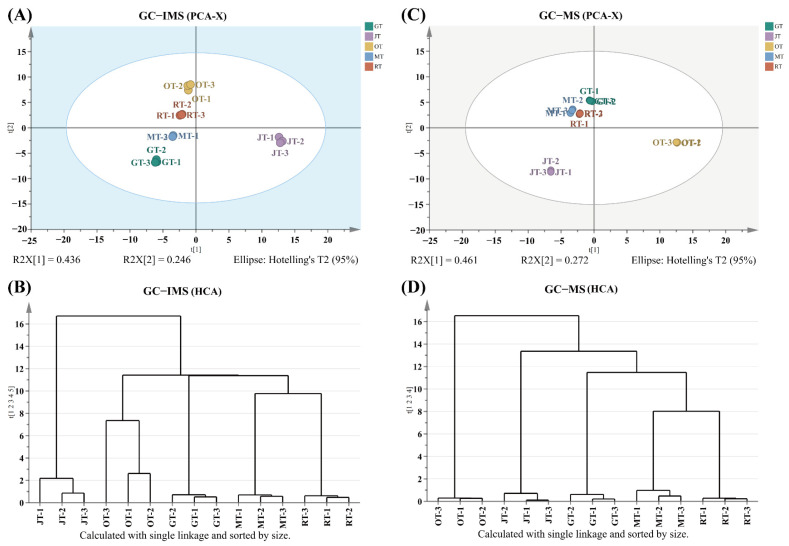
(**A**) PCA plot based on the results of GC–IMS; (**B**) HCA analysis based on the results of GC–IMS; (**C**) PCA plot based on the results of GC–MS; (**D**) HCA analysis based on the results of GC–IMS; (GT: green tea, JT: jasmine tea, OT: osmanthus tea, MT: michelia tea, RT: rose tea).

**Figure 5:**
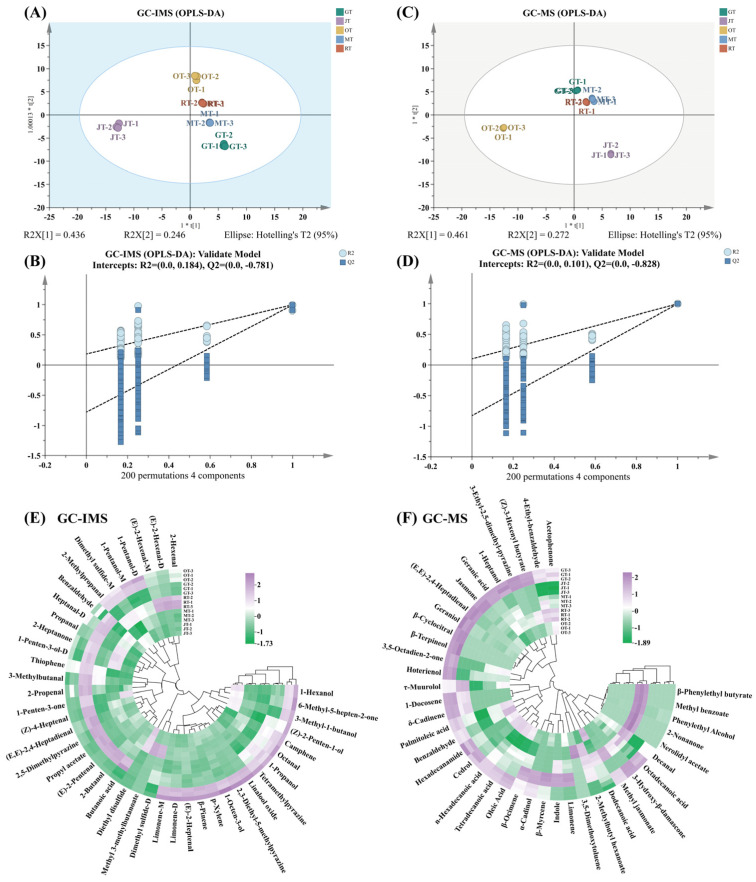
(**A**) OPLS–DA plot based on the results of GC–IMS; (**B**) a permutation test was performed to validate the OPLS–DA model established using the results of GC–IMS; (**C**) OPLS–DA plot based on the results of GC–MS; (**D**) a permutation test was performed to validate the OPLS–DA model established using the results of GC–MS; (**E**) heat map analysis of marker metabolites (VIP > 1) identified by means of GC–IMS; (**F**) heat map analysis of marker metabolites (VIP > 1) identified by means of GC–MS. (GT: green tea, JT: jasmine tea, OT: osmanthus tea, MT: michelia tea, RT: rose tea).

To elucidate the variances among the four types of scented teas and Jingshan green tea, principal component analysis (PCA) was utilized to assess the five tea samples. The compounds detected by means of GC–IMS and GC–MS, along with their corresponding peak intensities, were utilized for multivariate statistical analysis. As illustrated in Figure 4A,C, samples of tea scented with identical flower types were clustered together. The results of hierarchical clustering analysis (HCA) indicated that JT and OT did not cluster together with the other tea samples. This could be attributed to the relatively high number of unique compounds or substantial variations in the content of shared compounds present in JT and OT. Orthogonal partial least squares discriminant analysis (OPLS-DA) was conducted on the volatile compounds determined by means of GC–IMS and GC–MS (Figure 5A,C) to facilitate separation and profiling. The model parameters for GC–IMS (R^2^X = 0.944, R^2^Y = 0.989, Q^2^ = 0.978) and GC–MS (R^2^X = 0.992, R^2^Y = 0.998, Q^2^ = 0.996) exhibited a high explanatory variance (R^2^Y) and strong predictive power (Q^2^) in both scenarios. In addition, the permutation test with 200 cross-validations indicated that the OPLS–DA model was reliable. R^2^X and R^2^Y represent the explanatory power of the model for the X and Y matrices, respectively, while Q^2^ indicates the predictive ability of the model. The closer these three indicators are to 1, the more stable and reliable the model is considered to be. A Q^2^ value greater than 0.5 suggests an effective model, while a Q^2^ value exceeding 0.9 indicates an outstanding model. The results indicated that the OPLS–DA model constructed in this experiment exhibited neither overfitting nor underfitting (Figure 5B,D) in terms of performance, and it could therefore be utilized for the subsequent screening of differential volatile compounds.

The VIP (variable importance in projection) values in the OPLS–DA model serve as a measure of variable importance within the model. The VIP value reflects the contribution of a variable to the overall model fit and its classification ability. Generally, variables with VIP values greater than 1 are considered particularly important for the model. By analyzing VIP values, the most useful variables for classification can be identified. Based on the VIP values from the OPLS–DA model, further analysis was conducted to elucidate the differential volatile compounds among the various types of scented tea and Jingshan green tea. In the samples analyzed using GC–IMS and GC–MS, 51 and 40 differential volatile compounds with VIP > 1 were selected. The differences in the contents of these volatile compounds in different tea samples could be visually demonstrated through a heat map, as these compounds had a significant impact on the classification of tea. As shown in Figure 5E,F, purple represented concentrations above the average, while green represented concentrations below the average. The GC–IMS results indicated that the levels of 1-pentanol, dimethyl sulfide, limonene, (*E*)-2-heptenal, *β*-pinene, *p*-xylene, 1-octen-3-ol, 2,3-dethyl-5-methylpyrazine, linalool oxide, tetramethylpyrazine, and 2-methylpropanal were relatively high in OT (Figure 5E). In addition, the contents of 2-hexenal and (*E*)-2-hexenal were higher in RT. The concentrations of methyl 3-methylbutanoate, diethyl disulfide, benzaldehyde, propanal, 2-heptanone, 1-penten-3-ol, and dimethyl sulfide were comparatively elevated in MT. Among these differentiated compounds, JT contained relatively high levels of 1-penten-3-one, diethyl disulfide, 1-hexanol, and 3-methyl-1-butanol. GT contained relatively high levels of volatile compounds such as benzaldehyde, heptanal, (*Z*)-4-heptenal, (*E*,*E*)-2,4-heptadienal, and 2-butanol. Despite Jingshan green tea having served as the raw material for the four types of scented tea, the subsequent drying process resulted in a diminished concentration of these compounds in the scented teas.

Volatile compounds with VIP values greater than 1 are typically considered particularly important for the model and serve as signature metabolites for sample classification. As shown in Figure 5, multivariate statistical analysis of the volatile compounds detected via GC–MS revealed the presence of certain marker metabolites [24]. For instance, in JT, relatively high levels of limonene, indole, *β*-myrcene, *α*-cadinol, *β*-ocimene, *τ*-muurolol, 1-docosene, and *δ*-cadinene were identified. MT exhibited relatively high levels of *β*-phenylethyl butyrate, methyl benzoate, phenylethyl alcohol, 2-nonanone, nerolidyl acetate, decanal, octadecanoic acid, methyl jasmonate, dodecanoic acid, and 2-methylbutyl hexanoate among the volatile compounds with VIP > 1. The levels of acetophenone, 4-ethyl-benzaldehyde, (*Z*)-3-hexenyl butyrate, 2-methylbutyl hexanoate, and 3,5-dimethoxytoluene in RT exhibited relatively high concentrations. OT exhibited relatively high levels of dodecanoic acid, oleic acid, tetradecanoic acid, n-hexadecanoic acid, cedrol, hexadecanamide, benzaldehyde, palmitoleic acid, and acetophenone among the selected differential volatile metabolites.

### 3.3. Screening for Characteristic Volatile Compounds

#### Key Aroma Compound Analysis Based on ROAV

**Figure 6:**
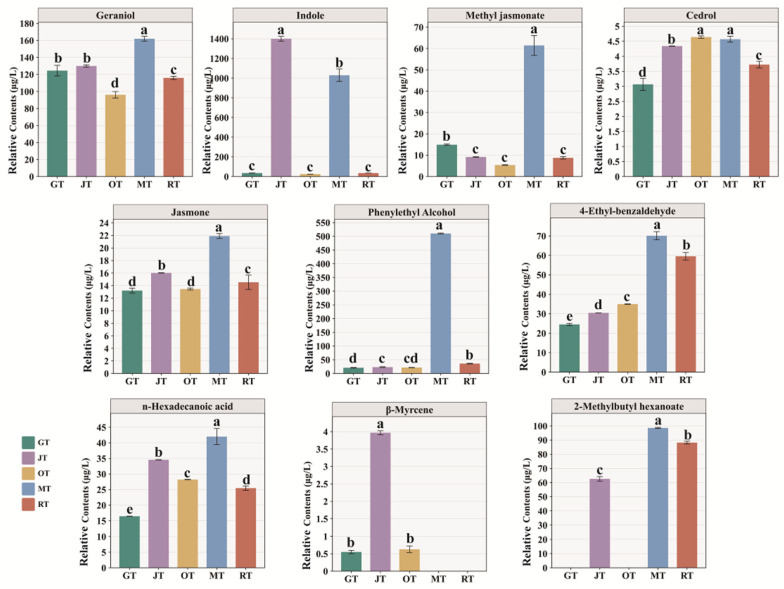
Relative content of key aroma compounds in different tea samples (mean ± SD). The different letters on the bar chart indicate that the content of compounds was significantly different (*p* < 0.05). (GT: green tea, JT: jasmine tea, OT: osmanthus tea, MT: michelia tea, RT: rose tea.).

The contribution of volatile compounds in tea samples to aroma is determined not only by their concentration but also by their odor threshold [25]. In this study, decanoic acid ethyl ester was used as an internal standard for the relative quantification of the compounds detected by means of GC–MS. Therefore, the ROAV was employed to preliminarily assess the contribution of individual compounds to the overall aroma. The fragrance contribution of volatile compounds can be assessed by calculating the relative odor activity value (ROAV), where a ROAV exceeding 1 signifies a substantial olfactory contribution [26]. The comprehensive aromatic contribution of volatile compounds to various scented tea samples was determined by employing ROAVs in this study (Table 1). The selection comprised ten key aroma compounds (VIP > 1, *p* < 0.05, ROAV > 1) [27], specifically geraniol, indole, methyl jasmonate, cedrol, jasmone, phenylethyl alcohol, 4-ethyl-benzaldehyde, n-hexadecanoic acid, *β*-myrcene, and 2-methylbutyl hexanoate. The ROAVs of these key aroma compounds and their respective contents in various tea samples are presented in Table 1 and Figure 6. Geraniol is a key aroma compound in tea, particularly floral green tea [28], and is generated through the hydrolysis of glycosidic substances caused by tissue damage during tea processing [4]. Geraniol is naturally present in *Jasminum sambac* and serves as the primary odor active compound, giving the tea floral characteristics [23]. In *Osmanthus fragrans* and osmanthus black tea, geraniol is also recognized as a key compound with high aroma activity value, and it also possesses a coconut-like flavor [7]. In previous reports, geraniol was also identified in the fresh flowers of *Michelia alba* [29]. Furthermore, geraniol is a primary component of both rose essential oil and rose flowers, making a significant contribution to the fragrance of roses [30,31]. Hence, geraniol was naturally present in all four types of flowers, as well as the GT, and it served as a significant aroma component in both the flowers and scented teas. However, not all scented teas exhibited a higher geraniol content compared to that of GT, which may have been due to the high-temperature drying process following scenting.

Indole is present in various plants and serves as a characteristic aroma compound in certain types of green tea [32,33]. Indole exhibits distinct aromatic characteristics across varying concentrations, manifesting a “floral fragrance” at lower concentrations and an “unpleasant odor” at higher concentrations [34]. The content of indole in JT and MT was significantly higher than that in the other tea samples, making it the characteristic aroma compound of *Jasminum sambac* and *Michelia alba*. Additionally, the levels of methyl jasmonate and phenylethyl alcohol in MT were significantly higher compared to those in the other tea samples. Methyl jasmonate has been detected in various types of tea and plays a significant role in the formation of tea aroma [35,36]. It is a volatile compound with floral characteristics. The presence of phenylethyl alcohol is also widespread [37,38], as it is a commonly found volatile compound with floral and fruity fragrances [39]. Cedrol is a volatile compound with a woody fragrance [40]. Jasmone, 4-ethyl-benzaldehyde, and n-hexadecanoic acid were detected in all five tea samples, with higher levels found in the scented teas compared to those in GT. These findings suggested that during scenting, all four types of fresh flowers released these key aroma compounds, which were subsequently absorbed by the GT. Jasmone and indole exhibit a characteristic floral fragrance [41], whereas n-hexadecanoic acid has a creamy aroma. In addition, *β*-myrcene possesses a fruity fragrance and was also one of the characteristic aroma compounds found in JT [42]. 2-Methylbutyl hexanoate has a fruity aroma, similar to that of pear or apple, and was more abundant in JT, MT, and RT than in GT and OT, suggesting that flower tea scenting with *Jasminum sambac*, *Michelia alba*, and *Rosa rugosa* is favorable for the development of a more intense fruity aroma.

The characteristic aromas of different tea samples were generated by the combined action of multiple aroma-active compounds [43]. The fresh flowers used for processing scented teas inherently possess an aroma. Despite the presence of numerous shared volatile compounds among the various scented teas, variations in the concentration of these common substances and the existence of unique compounds specific to each type of scented tea resulted in distinct aromatic profiles for the various scented teas. The sweetness of OT was speculated to be derived from *γ*-decalactone, (*E*)-*β*-ionone, and other compounds with sweetness [22]. The sweetness of MT was primarily attributed to the key aroma compounds, such as phenylethyl alcohol [44]. However, there may be synergistic or antagonistic effects between the aroma compounds of flowers and tea. Therefore, further investigation is warranted to optimize the scenting process and achieve a harmonious coordination of tea and floral aromas.

## 4. Conclusions

In this study, GC–IMS was employed to detect and analyze the volatile compounds of four types of scented teas, as well as the green tea raw material used in their processing. By visualizing fingerprint chromatograms, it was possible to rapidly distinguish the differences between the four types of scented teas. The results indicated significant variations in the compound contents among the different samples of scented tea. The rapid identification of different types of scented teas has also been explored [45]. Furthermore, the volatile compounds in various scented teas were detected and identified by means of GC–MS. The combination of GC–IMS and GC–MS detection with multivariate statistical analysis allowed for the identification of marker metabolites among the volatile compounds. Among the selected key aroma compounds, geraniol played a pivotal role in shaping the characteristic aroma profile of each sample [4,7,28,29]. Moreover, key aroma compounds such as indole, methyl jasmonate, phenylethyl alcohol, and *β*-myrcene significantly contributed to the aroma profiles of JT and MT. In previous research, these compounds have been identified as important aroma compounds with floral, fruity, or sweet characteristics [22,35]. In this research, they were also recognized as key aroma compounds that contribute to the characteristic aromas of JT and MT. In addition, OT contained high levels of *γ*-decalactone and (*E*)-*β*-ionone, which are the key aroma compounds contributing to the prominent sweetness of osmanthus tea [22,46].

The analysis conducted using GC–IMS and GC–MS also revealed the presence of certain volatile compounds that were unique to or present in relatively high concentrations in specific types of scented teas. These compounds included characteristic volatile components of scented teas, such as 3,5-dimethoxytoluene in RT [17,31], methyl benzoate and *β*-phenylethyl butyrate in MT, methyl anthranilate and methyl salicylate in JT [21], and *γ*-nonanolactone, dihydro-*β*-ionol, camphor, menthone, and piperitone in OT [22,46], among others. These volatile compounds are also important components of various scented teas. Furthermore, the sensory evaluation results also confirmed that the various scented tea samples had different aroma components. The findings of this study indicated that the aroma profiles and volatile compound fingerprints of the different types of scented teas were distinct.

In summary, several characteristic compounds, including linalool, geraniol, jasmone, methyl jasmonate, hexadecanoic acid, 4-ethyl-benzaldehyde, cedrol, and indole, were commonly shared among all samples. The main characteristic volatile compounds of JT were benzyl acetate, *β*-myrcene, ethyl benzoate, *δ*-decalactone, 2-methylbutyl hexanoate, (*E*)-3-hexenyl benzoate, and oplopanone. The main characteristic volatile compounds of OT were (*E*)-*β*-ionone, *γ*-decalactone, dihydro-*β*-ionol, *γ*-nonanolactone, menthone, camphor, and piperitone. Certain volatile compounds, such as phenylethyl alcohol, 2-methylbutyl hexanoate, and methyl benzoate, were characteristic aromatic components of MT. The characteristic compounds of RT included 3,5-dimethoxytoluene, (*E*)-*β*-ionone, and 2-methylbutyl hexanoate. This study aimed to characterize the aroma profiles of four traditional scented teas with a base of green tea, thereby enhancing our understanding of the volatile components present in different types of scented teas. Based on this study, it will be possible to further explore and optimize the processing techniques for various scented teas by leveraging the characteristic volatile compounds of different flowers in order to enhance the quality of the scented teas. These results establish a theoretical foundation for the scenting process of green tea.

## Figures and Tables

**Table 1:** ROAVs of key volatile compounds in different tea samples detected via GC–MS.

No.	CAS	Compound	RI ^a^	Threshold (μg·L^−1^) ^b^	Aroma Description ^c^	Identification Basis ^d^	ROAV ^e^				
							GT	JT	OT	MT	RT
1	100-52-7	Benzaldehyde	972	150	Fruity, almond-like	MS/RI	0.01	0.01	0.03	n.d.	0.02
2	111-70-6	1-Heptanol	985	425	Green, sweet	MS/RI	0.00	n.d.	n.d.	0.00	0.00
3	123-35-3	*β*-Myrcene	1002	1.2	Geranium-like	MS/RI/Std	0.41	2.60	0.60	n.d.	n.d.
4	4313-03-5	(*E*,*E*)-2,4-Heptadienal	1023	10	Fatty, nutty, hay, green	MS/RI	0.98	n.d.	0.33	0.20	0.22
5	138-86-3	Limonene	1038	200	Citrus, lemon, orange-like	MS/RI	n.d.	0.01	0.01	0.00	n.d.
6	13877-91-3	*β*-Ocimene	1059	34	Citrus	MS/RI	0.02	0.07	0.02	0.04	n.d.
7	98-86-2	Acetophenone	1077	65	Sweet	MS/RI	0.09	0.06	0.18	0.15	0.23
8	13360-65-1	3-Ethyl-2,5-dimethyl-pyrazine	1090	25	Earthy	MS/RI	0.02	n.d.	n.d.	n.d.	0.01
9	821-55-6	2-Nonanone	1102	41	Earthy, herbal	MS/RI	n.d.	n.d.	n.d.	0.03	n.d.
10	38284-27-4	3,5-Octadien-2-one	1104	150	n.f.	MS/RI	0.02	n.d.	0.03	n.d.	0.02
11	93-58-3	Methyl benzoate	1105	73	Violet-like, floral	MS/RI	n.d.	n.d.	n.d.	0.21	n.d.
12	7299-41-4	*β*-Terpineol	1107	330	n.f.	MS/RI	0.01	n.d.	n.d.	n.d.	n.d.
13	29957-43-5	Hoterienol	1115	110	Fresh, floral, fruity	MS/RI	0.12	n.d.	0.09	0.09	0.11
14	1960/12/8	Phenylethyl alcohol	1125	140	Floral, honey-like	MS/RI	0.13	0.13	0.18	2.48	0.24
15	16491-36-4	(*Z*)-3-Hexenyl butyrate	1195	500	Fruity	MS/RI	0	n.d.	n.d.	n.d.	0
16	112-31-2	Decanal	1213	9.3	Citrus-like	MS/RI	0.07	n.d.	0.17	0.27	0.16
17	4748-78-1	4-Ethyl-benzaldehyde	1222	40	Almond-like	MS/RI	0.54	0.6	1	1.19	1.41
18	432-25-7	*β*-Cyclocitral	1227	3	Fruity	MS/RI	0.78	n.d.	n.d.	n.d.	n.d.
19	2601-13-0	2-Methylbutyl hexanoate	1229	30	n.f.	MS/RI	n.d.	1.64	n.d.	2.23	2.79
20	106-24-1	Geraniol	1263	1.1	Rose-like, citrus-like	MS/RI/Std	100	92.86	100	100	100
21	4179-19-5	3,5-Dimethoxytoluene	1275	n.f.	Sweet	MS/RI	-	-	-	-	-
22	120-72-9	Indole	1306	11	Fecal, mothball-like	MS/RI/Std	2.87	100	2.55	63.58	3.05
23	4698/8/2	Geranic acid	1372	10000	Sour	MS/RI	0	0	n.d.	0	0
24	488-10-8	Jasmone	1406	7	Celery-like	MS/RI	1.67	1.8	2.2	2.12	1.97
25	103-52-6	*β*-Phenylethyl butyrate	1457	376	Fruity, sweet	MS/RI	n.d.	n.d.	n.d.	0.05	n.d.
26	483-76-1	*δ*-Cadinene	1533	n.f.	Fresh, woody	MS/RI	-	-	-	-	-
27	143-07-7	Dodecanoic acid	1581	100	n.f.	MS/RI	0.1	0.28	0.44	0.42	0.21
28	77-53-2	Cedrol	1612	0.5	Woody, sweet	MS/RI	5.42	6.82	10.62	6.2	7.07
29	102488-09-5	3-Hydroxy-*β*-damascone	1626	n.f.	n.f.	MS/RI	-	-	-	-	-
30	19912-62-0	*τ*-Muurolol	1654	n.f.	Herbal	MS/RI	-	-	-	-	-
31	1211-29-6	Methyl jasmonate	1658	3	Floral, jasmine, green	MS/RI	4.4	2.4	2.06	13.9	2.78
32	481-34-5	*α*-Cadinol	1667	n.f.	Herbal, woody	MS/RI	-	-	-	-	-
33	2306-78-7	Nerolidyl acetate	1683	n.f.	Floral	MS/RI	-	-	-	-	-
34	544-63-8	Tetradecanoic acid	1769	10	Coconut-like	MS/RI	0.43	0.51	0.84	0.63	n.d.
35	373-49-9	Palmitoleic acid	1944	11	n.f.	MS/RI	0.4	0.55	0.8	n.d.	0.66
36	1957/10/3	n-Hexadecanoic acid	1963	10	Creamy	MS/RI	1.45	2.72	3.23	2.85	2.42
37	112-80-1	Oleic acid	2136	200	Fatty	MS/RI	0.02	0.02	0.03	0.03	n.d.
38	1957/11/4	Octadecanoic acid	2155	100	n.f.	MS/RI	0.03	0.05	0.05	0.07	0.06

Note: ^a^ Retention index (RI), “n.d.” means not detected. ^b^ All odor thresholds were obtained from: Leibniz-LSB@TUM Odorant Database (https://www.leibniz-lsb.de/datenbanken/leibniz-lsbtum-odorant-database/start) (accessed on 20 June 2024), [11,22,25,28] (n.f., data were not found in the literature). ^c^ Aroma description reported in the literature [1,4,19,25] and http://www.thegoodscentscompany.com/search2.html (accessed on 20 June 2024). (n.f., data were not found in the literature). ^d^ Methods of identification: MS, odorants were identified from mass spectra; RI, retention indices; and Std, reference compounds. For MS/MS spectral similarity, this was carried out using the dot product, inverse dot product, and matching fragmentation ratios with a weighting ratio of 1:1:1, and compounds with > 80% similarity were identified. ^e^ Relative aroma activity value (ROAV); green tea (GT), jasmine tea (JT), osmanthus tea (OT), michelia tea (MT), and rose tea (RT).

## Data Availability

The original contributions presented in the study are included in the article/Supplementary Materials, further inquiries can be directed to the corresponding authors.

## Supplementary Materials

The following supporting information can be downloaded at: https://www.mdpi.com/article/10.3390/foods13172653/s1, Table S1. Relative contents of volatile compounds in different tea samples analyzed by GC–IMS. Table S2. Volatile compounds identified in different tea samples by SBSE–GC–MS.
