# Supplementary material for: Comparison of Volatile Compounds in Jingshan Green Tea Scented with Different Flowers Using GC-IMS and GC-MS Analyses
Source: Foods. 2024 Aug 23;13(17):2653. doi: 10.3390/foods13172653 (PMC11394657; doi:10.3390/foods13172653)
Supplement: Supplementary file 1 [file foods-13-02653-s001.zip › foods-3139586-supplementary.pdf]

Table S1. The relative contents of volatile compounds in different tea samples detected by GC–IMS.

No	CAS	Compounds	Category	RI[a]	Drift Time/ms	Drift Time/ms	Peak intensity[b]				
							GT	JT	OT	MT	RT
1	75-18-3	Dimethyl sulfide-M	Others	794	226	0.96	5244.30±34.64^{d}	5809.52±5.28^{c}	6250.51±131.65^{a}	4626.35±52.00^{e}	6024.26±5.56^{b}
2	75-18-3	Dimethyl sulfide-D	Others	796	227	1.12	3083.77±22.36^{b}	2694.88±6.66^{c}	2129.79±153.14^{d}	4182.72±26.97^{a}	2682.43±41.70^{c}
3	123-38-6	Propanal	Aldehydes	819	239	1.14	5482.51±13.35^{a}	3229.00±9.64^{d}	3724.87±75.98^{c}	5022.78±29.75^{b}	3186.55±13.39^{d}
4	78-84-2	2-Methylpropanal	Aldehydes	830	245	1.28	250.19±0.42^{d}	584.33±3.06^{c}	704.20±21.68^{a}	248.76±1.26^{d}	606.35±5.34^{b}
5	67-64-1	Acetone	Ketones	841	251	1.11	7026.90±12.19^{b}	5737.05±2.09^{d}	5747.16±115.89^{d}	7213.85±4.28^{a}	6607.22±9.71^{c}
6	79-20-9	Methyl acetate	Esters	855	259	1.19	329.72±5.51^{d}	1132.75±4.32^{a}	860.33±58.39^{b}	483.37±4.98^{c}	478.06±2.04^{c}
7	107-02-8	2-Propenal	Aldehydes	866	265	1.06	376.55±7.80^{a}	215.68±47.74^{bc}	234.91±39.36^{b}	176.38±3.06^{c}	229.48±10.18^{bc}
8	123-72-8	Butanal	Aldehydes	891	279	1.28	1220.32±5.85^{a}	388.93±11.93^{d}	868.99±63.86^{b}	819.48±15.82^{b}	751.82±5.50^{c}
9	141-78-6	Ethyl acetate	Esters	900	285	1.34	2233.05±25.01^{b}	8113.85±7.93^{a}	2096.77±131.20^{c}	2073.44±64.79^{c}	2165.79±18.36^{bc}
10	105-57-7	Acetal	Aldehydes	909	291	1.04	4787.48±16.36^{d}	1380.98±7.12^{e}	5892.70±14.53^{b}	5631.15±22.14^{a}	5523.21±7.34^{c}
11	78-93-3	2-Butanone	Ketones	921	298	1.25	1663.71±32.60^{b}	2680.58±7.97^{a}	820.45±42.79^{d}	1319.50±10.67^{c}	1273.36±14.35^{c}
12	590-86-3	3-Methylbutanal	Aldehydes	933	307	1.41	5435.18±38.93^{a}	4496.31±3.92^{c}	4563.07±109.09^{c}	4566.34±21.59^{c}	5050.26±13.87^{b}
13	64-17-5	Ethanol	Alcohols	951	319	1.13	13460.45±263.29^{c}	39621.78±39.88^{a}	14368.52±159.44^{b}	12124.52±25.45^{d}	12353.64±80.45^{d}
14	109-60-4	Propyl acetate	Esters	997	353	1.48	139.95±6.08^{a}	61.20±2.26^{b}	24.92±4.35^{d}	38.74±2.57^{c}	28.59±0.62^{d}
15	110-62-3	Pentanal	Aldehydes	1005	361	1.42	6008.20±128.29^{b}	1572.38±3.78^{d}	6276.77±81.36^{a}	5996.57±40.54^{b}	5360.89±13.51^{c}
16	-	10	-	1008	364	1.51	242.91±12.23^{b}	159.15±1.86^{cd}	171.98±16.98^{d}	267.04±4.00^{a}	181.08±1.43^{c}
17	556-24-1	Methyl 3-methylbutanoate	Esters	1028	386	1.53	239.31±9.10^{c}	243.03±0.75^{c}	290.63±21.50^{c}	10295.07±54.78^{a}	393.23±10.12^{b}
18	110-02-1	Thiophene	Others	1035	393	1.04	5884.54±46.07^{a}	2083.91±21.29^{d}	4477.22±110.12^{c}	1757.19±9.88^{e}	4703.79±27.74^{b}
19	-	11	-	1035	394	1.18	389.67±19.40^{d}	2783.45±29.70^{a}	531.43±29.86^{c}	223.04±3.03^{e}	615.53±4.94^{b}
20	-	13	-	1040	399	1.26	163.63±3.77^{d}	266.55±3.65^{c}	329.13±39.44^{b}	1241.63±7.27^{a}	260.98±13.22^{c}
21	1629-58-9	1-Penten-3-one	Ketones	1045	406	1.08	573.78±2.99^{a}	537.68±6.38^{b}	265.06±6.30^{c}	253.10±15.77^{c}	195.95±5.06^{d}
22	78-92-2	2-Butanol	Alcohols	1045	406	1.31	1293.05±24.01^{a}	405.97±6.43^{c}	286.62±50.04^{d}	366.24±12.90^{c}	505.84±28.26^{b}
23	71-23-8	1-Propanol	Alcohols	1058	421	1.11	445.39±7.84^{b}	258.31±1.09^{e}	508.57±46.57^{a}	302.27±1.89^{c}	266.81±3.20^{cd}
24	-	9	-	1058	421	1.16	114.28±9.42^{b}	93.92±0.66^{c}	135.93±15.10^{a}	91.73±2.66^{c}	68.53±0.91^{d}
25	105-54-4	Ethyl butanoate-M	Esters	1059	421	1.2	25.57±1.56^{b}	403.54±6.82^{a}	18.04±1.90^{c}	24.67±1.51^{b}	17.72±0.53^{c}
26	105-54-4	Ethyl butanoate-D	Esters	1059	421	1.56	162.80±10.22^{d}	1083.59±17.51^{a}	197.35±12.87^{c}	309.19±0.83^{b}	114.48±8.47^{e}
27	79-92-5	Camphene	Hydrocarbons	1068	433	1.2	244.15±10.81^{b}	79.64±4.26^{d}	317.59±30.87^{a}	191.04±2.52^{c}	104.32±5.28^{d}
28	-	8	-	1073	440	1.41	111.12±4.95^{ab}	57.26±3.17^{c}	127.59±26.66^{a}	104.83±9.18^{ab}	99.56±3.32^{b}
29	624-92-0	Dimethyl disulfide	Others	1088	459	1.13	177.77±5.85^{b}	139.37±6.21^{b}	423.79±192.97^{a}	220.56±6.61^{b}	277.47±5.95^{ab}
30	123-86-4	Butyl acetate-M	Esters	1092	465	1.24	505.40±7.53^{b}	1081.69±17.64^{a}	155.74±74.36^{d}	328.78±9.60^{c}	271.16±4.13^{c}
31	123-86-4	Butyl acetate-D	Esters	1093	465	1.62	79.51±2.48^{b}	463.3±9.67^{a}	37.85±11.02^{d}	55.73±2.00^{c}	51.20±0.66^{c}
32	-	6	-	1096	470	1.39	863.07±14.56^{a}	347.61±12.39^{d}	295.43±43.60^{d}	448.54±6.57^{b}	405.62±3.71^{c}
33	-	7	-	1097	470	1.82	128.11±6.68^{a}	51.95±1.79^{b}	51.90±3.07^{b}	50.74±6.62^{b}	46.69±2.30^{b}
34	66-25-1	Hexanal-D	Aldehydes	1105	484	1.56	4464.39±21.32^{a}	797.30±11.57^{e}	2107.33±248.10^{d}	3143.75±11.07^{b}	2654.34±44.09^{c}
35	66-25-1	Hexanal-M	Aldehydes	1106	485	1.27	2783.29±34.46^{a}	1781.51±22.36^{c}	2691.54±69.58^{b}	2719.12±6.14^{ab}	2762.80±15.86^{a}
36	78-83-1	2-Methyl-1-propanol	Alcohols	1111	495	1.18	325.85±8.11^{c}	589.69±8.69^{a}	554.34±100.02^{a}	347.73±7.29^{c}	431.95±5.38^{b}
37	584-02-1	3-Pentanol	Alcohols	1112	496	1.42	169.44±5.64^{e}	1077.35±16.29^{a}	299.41±58.68^{d}	632.00±17.65^{b}	402.33±7.32^{c}
38	-	5	-	1113	497	1.11	178.77±1.19^{d}	95.90±1.60^{e}	320.47±31.84^{c}	485.06±4.38^{a}	386.57±2.79^{b}
39	-	4	-	1119	508	1.09	197.05±4.52^{c}	285.54±2.71^{b}	190.69±18.83^{c}	320.91±5.55^{a}	199.53±6.74^{c}

No	CAS	Compounds	Category	RI[a]	Drift Time/ms	Drift Time/ms	Peak intensity[b]				
							GT	JT	OT	MT	RT
40	-	14	-	1126	520	1.2	63.90±5.22^{c}	80.05±4.29^{b}	54.07±5.87^{d}	86.29±0.97^{b}	98.29±1.58^{a}
41	1576-87-0	(*E*)-2-Pentenal	Aldehydes	1130	527	1.11	170.86±7.67^{a}	60.01±1.41^{b}	34.18±2.97^{d}	46.30±2.53^{c}	24.27±0.74^{e}
42	127-91-3	*β*-Pinene	Hydrocarbons	1134	535	1.22	94.06±7.94^{bc}	104.21±2.32^{b}	254.86±19.81^{a}	102.61±3.51^{b}	76.95±3.90^{c}
43	106-42-3	*p*-Xylene	Hydrocarbons	1136	537	1.09	51.50±2.47^{c}	73.23±1.24^{b}	219.67±23.47^{a}	39.22±0.62^{cd}	28.86±1.36^{d}
44	120-92-3	Cyclopentanone-M	Ketones	1147	558	1.12	9969.96±273.54^{a}	5794.02±203.57^{c}	6762.72±1120.48^{c}	7873.31±457.23^{b}	8086.00±412.51^{b}
45	120-92-3	Cyclopentanone-D	Ketones	1147	558	1.34	3744.69±74.15^{b}	3225.74±16.72^{c}	3850.93±101.94^{b}	4021.98±55.34^{a}	4080.89±39.20^{a}
46	-	2	-	1153	571	1.4	1060.06±16.95^{a}	718.76±18.31^{b}	688.41±181.27^{b}	1070.54±37.52^{a}	932.93±39.77^{a}
47	-	3	-	1153	571	1.45	543.74±19.56^{c}	1012.68±2.80^{b}	590.39±125.83^{c}	1390.44±8.98^{a}	1085.94±19.88^{b}
48	71-36-3	1-Butanol-M	Alcohols	1163	589	1.18	847.36±22.02^{b}	1617.98±16.53^{a}	767.30±95.04^{bc}	751.41±6.90^{c}	698.59±5.37^{c}
49	71-36-3	1-Butanol-D	Alcohols	1163	590	1.38	141.61±7.42^{b}	1459.09±15.18^{a}	114.56±25.07^{cd}	121.94±4.40^{bc}	93.09±2.85^{d}
50	616-25-1	1-Penten-3-ol-M	Alcohols	1178	622	0.94	2915.47±29.20^{a}	2025.92±24.71^{e}	2455.74±35.19^{d}	2738.31±10.50^{b}	2607.76±7.93^{c}
51	616-25-1	1-Penten-3-ol-D	Alcohols	1178	622	1.35	2585.06±63.64^{a}	1801.13±26.33^{d}	1845.90±213.28^{d}	2442.83±5.77^{a}	1864.84±47.80^{d}
52	110-43-0	2-Heptanone	Ketones	1190	648	1.26	112.11±3.94^{a}	44.48±1.47^{c}	62.70±14.61^{b}	109.58±2.99^{a}	60.41±4.03^{b}
53	95-47-6	*o*-Xylene	Hydrocarbons	1193	653	1.08	79.48±3.48^{a}	65.89±3.88^{c}	73.98±3.17^{b}	66.48±1.70^{c}	68.01±1.08^{c}
54	-	1	-	1196	658	1.26	103.42±5.32^{a}	54.66±1.79^{d}	82.23±9.97^{b}	95.03±0.69^{a}	71.72±2.42^{c}
55	-	12	-	1199	662	1.04	205.07±14.78^{d}	1097.00±9.10^{a}	240.48±31.98^{c}	295.62±3.85^{b}	247.43±5.38^{c}
56	111-71-7	Heptanal-D	Aldehydes	1200	662	1.7	702.83±8.55^{a}	200.57±3.34^{c}	428.98±81.19^{b}	464.86±8.33^{b}	239.51±7.89^{c}
57	111-71-7	Heptanal-M	Aldehydes	1200	663	1.34	1438.96±9.59^{a}	640.92±6.63^{d}	1051.79±76.88^{b}	1066.32±2.97^{b}	856.66±14.47^{c}
58	110-81-6	Diethyl disulfide	Others	1208	674	1.14	34.87±1.12^{c}	188.08±10.55^{a}	24.95±2.27^{d}	169.20±2.35^{b}	24.25±3.46^{d}
59	138-86-3	Limonene-M	Hydrocarbons	1209	675	1.22	87.97±4.88^{b}	67.10±3.09^{c}	199.23±20.59^{a}	72.19±2.74^{bc}	46.75±2.46^{d}
60	138-86-3	Limonene-D	Hydrocarbons	1210	676	1.3	74.21±1.78^{b}	50.92±2.56^{b}	217.05±28.05^{a}	75.44±1.48^{b}	53.26±5.38^{b}
61	107-86-8	3-Methyl-2-butenal	Aldehydes	1218	688	1.09	53.36±3.83^{a}	39.38±2.40^{b}	35.39±9.09^{bc}	36.76±1.48^{bc}	29.81±2.54^{c}
62	505-57-7	2-Hexenal	Aldehydes	1220	691	1.18	36.46±0.93^{c}	39.62±2.85^{c}	57.43±2.92^{b}	35.75±3.25^{c}	85.93±5.49^{a}
63	123-51-3	3-Methyl-1-butanol	Alcohols	1223	695	1.24	70.92±10.40^{e}	468.63±4.63^{b}	634.86±65.16^{a}	221.96±4.78^{c}	145.26±2.25^{d}
64	6728-26-3	(*E*)-2-Hexenal-M	Aldehydes	1234	710	1.18	428.58±11.55^{c}	110.49±1.94^{e}	557.20±46.59^{b}	227.76±1.19^{d}	1320.78±31.78^{a}
65	6728-26-3	(*E*)-2-Hexenal-D	Aldehydes	1234	711	1.52	57.17±2.99^{c}	20.36±2.62^{d}	94.43±16.70^{b}	26.64±4.07^{cd}	585.09±35.44^{a}
66	108-65-6	1-Methoxy-2-propanol acetate	Esters	1243	724	1.14	44.93±1.81^{b}	85.50±5.42^{a}	20.71±0.43^{d}	23.50±1.08^{d}	28.65±1.35^{c}
67	3777-69-3	2-Pentylfuran	Heterocyclics	1247	730	1.25	41.39±2.54^{a}	39.95±2.32^{a}	31.94±4.57^{c}	37.70±1.48^{ab}	34.38±0.62^{bc}
68	6728-31-0	(*Z*)-4-Heptenal	Aldehydes	1259	747	1.15	128.70±0.32^{a}	66.50±3.77^{c}	47.26±3.88^{d}	75.78±4.68^{b}	36.23±0.71^{e}
69	71-41-0	1-Pentanol-D	Alcohols	1268	762	1.51	504.43±5.03^{d}	741.12±8.66^{c}	1484.73±113.03^{a}	820.79±6.99^{c}	966.75±12.85^{b}
70	71-41-0	1-Pentanol-M	Alcohols	1269	762	1.26	1587.44±39.57^{d}	1720.76±20.34^{a}	2537.48±66.75^{c}	2017.56±5.09^{b}	2179.53±20.20^{e}
71	763-32-6	3-Methyl-3-buten-1-ol	Alcohols	1277	775	1.2	28.98±5.49^{b}	250.53±7.53^{c}	73.74±9.04^{d}	37.23±2.64^{d}	33.16±0.67^{a}
72	99-87-6	*p*-Cymene	Hydrocarbons	1297	809	1.16	134.08±1.82^{b}	176.28±4.08^{c}	126.40±14.89^{d}	98.48±1.51^{e}	79.55±4.33^{a}
73	513-86-0	Acetoin-M	Ketones	1302	816	1.06	409.29±6.07^{d}	1364.83±10.14^{a}	957.08±68.55^{b}	491.60±3.64^{c}	472.22±3.42^{c}
74	513-86-0	Acetoin-D	Ketones	1302	817	1.33	38.90±5.20^{c}	276.79±5.84^{a}	155.82±32.58^{b}	46.98±3.55^{c}	38.36±3.02^{c}

No	CAS	Compounds	Category	RI[a]	Drift Time/ms	Drift Time/ms	Peak intensity[b]				
							GT	JT	OT	MT	RT
75	124-13-0	Octanal	Aldehydes	1302	817	1.4	157.85±7.11b	107.85±3.07^{c}	198.75±24.70^{a}	137.61±10.13^{b}	92.83±2.10^{c}
76	116-09-6	1-Hydroxy-2-propanone-M	Ketones	1317	843	1.06	661.86±9.17^{cd}	994.14±20.74^{a}	637.27±29.18^{d}	671.74±11.16^{bc}	698.11±5.59^{b}
77	116-09-6	1-Hydroxy-2-propanone-D	Ketones	1318	845	1.24	67.02±1.89^{b}	228.34±6.51^{a}	62.74±9.56^{b}	70.27±1.14^{b}	68.44±2.87^{b}
78	18829-55-5	(*E*)-2-Heptenal	Aldehydes	1333	873	1.26	80.09±6.01^{c}	84.46±3.68^{c}	325.85±31.95^{a}	120.02±5.25^{b}	117.37±3.24^{b}
79	3681-71-8	(*Z*)-3-Hexenyl acetate-M	Esters	1333	874	1.32	185.57±11.39^{c}	3183.83±27.42^{a}	260.86±45.91^{b}	209.23±14.10^{c}	171.10±14.33^{c}
80	3681-71-8	(*Z*)-3-Hexenyl acetate-D	Esters	1333	874	1.82	216.98±9.66^{bc}	9493.05±40.57^{a}	191.85±11.10^{c}	218.73±11.85^{bc}	235.48±17.62^{b}
81	1576-95-0	(*Z*)-2-Penten-1-ol	Alcohols	1341	889	0.94	930.86±18.32^{a}	953.11±6.04^{a}	981.22±85.47^{a}	824.58±8.01^{b}	745.69±10.06^{c}
82	2445-76-3	Hexyl propionate	Esters	1344	893	1.42	154.08±7.25^{b}	194.76±17.72^{a}	139.95±22.72^{b}	140.00±8.85^{b}	143.84±26.03^{b}
83	110-93-0	6-Methyl-5-hepten-2-one	Ketones	1352	909	1.18	128.63±2.90^{c}	160.67±2.87^{b}	193.85±27.79^{a}	108.36±3.15^{cd}	95.99±1.15^{d}
84	123-32-0	2,5-Dimethylpyrazine	Heterocyclics	1353	912	1.11	101.99±0.95^{a}	59.77±0.69^{c}	57.48±4.77^{c}	67.03±5.49^{b}	55.49±0.95^{c}
85	111-27-3	1-Hexanol	Alcohols	1374	953	1.33	227.60±19.33^{b}	417.39±3.95^{a}	463.12±67.11^{a}	185.07±7.58^{b}	169.51±3.56^{b}
86	142-83-6	2,4-Hexadienal	Aldehydes	1389	986	1.09	75.69±3.92^{a}	79.60±5.00^{a}	57.86±3.14^{c}	68.70±2.21^{b}	58.00±2.30^{c}
87	928-96-1	(*Z*)-3-Hexenol-M	Alcohols	1406	1022	1.24	735.73±46.61^{b}	2981.99±45.79^{a}	723.02±63.95^{b}	544.20±13.41^{c}	451.48±3.26^{d}
88	124-19-6	Nonanal	Aldehydes	1406	1022	1.48	274.78±20.14^{c}	899.59±13.16^{a}	423.02±47.67^{b}	241.81±15.61^{cd}	194.54±17.19^{d}
89	928-96-1	(*Z*)-3-Hexenol-D	Alcohols	1406	1023	1.51	284.04±19.85^{c}	4914.60±68.84^{a}	382.03±48.53^{b}	264.62±24.56^{cd}	205.17±9.94^{d}
90	2548-87-0	(*E*)-2-Octenal	Aldehydes	1441	1102	1.34	91.57±7.59^{ab}	98.93±31.43^{ab}	110.12±23.40^{a}	78.86±2.93^{ab}	68.69±1.18^{b}
91	60047-17-8	Linalool oxide	Alcohols	1458	1143	1.27	106.15±11.78^{b}	139.90±15.61^{b}	1050.68±159.63^{a}	135.11±13.76^{b}	117.08±5.86^{b}
92	3268-49-3	Methional	Aldehydes	1478	1193	1.09	103.88±15.33^{bc}	131.84±11.23^{a}	113.76±8.60^{b}	91.89±3.11^{c}	87.92±6.71^{c}
93	16491-36-4	(*Z*)-3-Hexen-1-ol butanoate	Esters	1487	1218	1.43	115.70±17.08^{c}	620.52±20.08^{a}	177.55±13.09^{b}	116.00±17.88^{c}	102.91±7.29^{c}
94	3391-86-4	1-Octen-3-ol	Alcohols	1488	1221	1.17	69.56±7.15^{b}	94.99±9.94^{b}	216.98±42.62^{a}	81.91±4.09^{b}	85.38±6.93^{b}
95	1124-11-4	Tetramethylpyrazine	Heterocyclics	1489	1223	1.2	192.3±13.70^{b}	144.65±1.31^{c}	258.35±33.16^{a}	128.82±7.60^{c}	90.00±9.73^{d}
96	18138-04-0	2,3-Diethyl-5-methylpyrazine	Heterocyclics	1492	1232	1.27	78.84±11.37^{c}	279.97±56.66^{b}	1225.31±220.20^{a}	148.49±23.33^{bc}	106.11±1.93^{bc}
97	64-19-7	Acetic acid-M	Acids	1505	1266	1.06	3626.16±83.06^{a}	4294.36±443.39^{a}	4400.73±816.26^{a}	3877.92±228.91^{a}	4341.87±43.55^{a}
98	64-19-7	Acetic acid-D	Acids	1505	1266	1.16	314.73±8.61^{b}	483.81±91.22^{ab}	566.55±250.90^{a}	398.66±48.10^{ab}	508.42±11.67^{ab}
99	4313-03-5	(*E*,*E*)-2,4-Heptadienal	Aldehydes	1519	1304	1.2	394.38±21.12^{a}	132.14±3.95^{c}	162.63±19.35^{b}	187.80±9.53^{b}	110.13±9.74^{c}
100	104-76-7	2-Ethyl-1-hexanol	Alcohols	1538	1361	1.42	189.26±24.72^{a}	197.52±34.14^{a}	176.61±6.19^{a}	184.51±16.32^{a}	177.04±18.59^{a}
101	100-52-7	Benzaldehyde	Aldehydes	1551	1399	1.16	445.94±19.12^{a}	339.13±14.94^{b}	337.00±59.54^{b}	395.00±10.95^{a}	276.05±11.87^{c}
102	106-23-0	Citronellal	Aldehydes	1564	1437	1.23	272.46±11.01^{a}	213.31±29.11^{b}	218.87±36.55^{b}	217.10±29.42^{b}	159.84±11.76^{c}
103	79-09-4	Propanoic acid	Acids	1638	1687	1.12	416.11±46.35^{c}	1254.07±24.52^{a}	893.03±310.90^{b}	707.94±68.57^{b}	899.92±57.68^{b}
104	78-70-6	Linalool-M	Alcohols	1646	1715	1.23	653.51±40.52^{b}	32817.02±156.75^{a}	1061.36±450.29^{b}	750.55±8.43^{b}	697.11±79.52^{b}
105	78-70-6	Linalool-D	Alcohols	1646	1717	1.7	1704.77±60.96^{b}	9884.41±127.74^{a}	1733.07±24.93^{b}	1748.66±6.35^{b}	1730.75±26.78^{b}
106	562-74-3	4-Terpinenol	Alcohols	1717	2002	1.22	222.76±13.49^{c}	2496.92±38.27^{a}	231.42±40.17^{c}	414.06±12.35^{b}	266.55±75.66^{c}
107	107-92-6	Butanoic acid	Acids	1721	2021	1.17	887.70±80.72^{a}	445.64±32.51^{b}	298.71±45.75^{c}	498.92±31.19^{b}	449.39±30.08^{b}

No	CAS	Compounds	Category	RI[a]	Drift Time/ms	Drift Time/ms	Peak intensity[b]				
							GT	JT	OT	MT	RT
108	122-78-1	Benzeneacetaldehyde	Aldehydes	1809	2442	1.27	319.52±45.26^{b}	2196.72±81.93^{a}	374.94±64.81^{b}	341.82±61.55^{b}	362.17±3.47^{b}

Note: [a] Retention index (RI). [b] Green tea (GT), Jasmine tea (JT), Osmanthus tea (OT), Michelia tea (MT) and Rose tea (RT). Values in data with different letters (a–e) in the same row are significantly different ($p < 0.05$).

Table S2. Volatile compounds identified in different tea samples by SBSE–GC–MS.

No	RI[a]	CAS	Compounds	Formula	Category	Relative Concentration (μg/L)[b]				
						GT	JT	OT	MT	RT
1	811	66-25-1	Hexanal	$C_6H_{12}O$	Aldehydes	1.38±0.04^{b}	n.d.	3.07±0.01^{a}	n.d.	0.98±0.05^{c}
2	871	928-97-2	(*E*)-3-Hexen-1-ol	$C_6H_{12}O$	Alcohols	1.36±0.04^{b}	28.98±0.22^{a}	n.d.	n.d.	n.d.
3	885	111-27-3	1-Hexanol	$C_6H_{14}O$	Alcohols	1.10±0.16^{d}	3.10±0.06^{a}	2.61±0.20^{b}	1.63±0.04^{c}	1.71±0.13^{c}
4	914	111-71-7	Heptanal	$C_7H_{14}O$	Aldehydes	0.39±0.05^{c}	0.66±0.07^{b}	1.08±0.07^{a}	n.d.	n.d.
5	972	100-52-7	Benzaldehyde	C_7H_6O	Aldehydes	2.00±0.07^{c}	1.92±0.03^{c}	4.46±0.02^{a}	n.d.	3.31±0.08^{b}
6	985	111-70-6	1-Heptanol	$C_7H_{16}O$	Alcohols	1.06±0.11^{a}	n.d.	n.d.	1.21±0.10^{a}	0.81±0.09^{b}
7	993	3391-86-4	1-Octen-3-ol	$C_8H_{16}O$	Alcohols	4.87±0.32^{e}	6.19±0.20^{d}	34.39±0.12^{a}	11.61±0.33^{c}	14.45±0.19^{b}
8	997	3214-41-3	2,5-Octanedione	$C_8H_{14}O_2$	Ketones	2.75±0.12^{d}	3.90±0.29^{c}	4.83±0.49^{b}	6.37±0.15^{a}	5.28±0.48^{b}
9	999	110-93-0	6-Methyl-5-hepten-2-one	$C_8H_{14}O$	Ketones	1.99±0.08^{e}	3.39±0.04^{d}	6.67±0.02^{a}	4.39±0.14^{b}	3.62±0.09^{c}
10	1002	123-35-3	*β*-Myrcene	$C_{10}H_{16}$	Hydrocarbons	0.55±0.05^{b}	3.97±0.06^{a}	0.63±0.10^{b}	n.d.	n.d.
11	1020	3681-82-1	(*E*)-Hex-3-enol acetate	$C_8H_{14}O_2$	Esters	0.76±0.04^{b}	671.68±45.34^{a}	3.57±0.89^{b}	3.01±0.14^{b}	4.62±0.89^{b}
12	1023	4313-03-5	(*E*,*E*)-2,4-Heptadienal	$C_7H_{10}O$	Aldehydes	11.06±0.56^{a}	n.d.	2.87±0.00^{b}	2.92±0.14^{b}	2.29±0.10^{c}
13	1038	138-86-3	Limonene	$C_{10}H_{16}$	Hydrocarbons	n.d.	3.47±0.06^{a}	1.03±0.01^{b}	1.14±0.09^{b}	n.d.
14	1055	122-78-1	Benzeneacetaldehyde	C_8H_8O	Aldehydes	4.42±0.50^{d}	n.d.	11.64±0.04^{b}	13.57±0.09^{a}	10.20±0.13^{c}
15	1056	100-51-6	Benzyl alcohol	C_7H_8O	Alcohols	0.64±0.05^{c}	27.74±0.04^{a}	n.d.	n.d.	4.22±0.19^{b}
16	1059	13877-91-3	*β*-Ocimene	$C_{10}H_{16}$	Hydrocarbons	0.78±0.03^{c}	3.22±0.11^{a}	0.56±0.10^{d}	1.96±0.13^{b}	n.d.
17	1077	98-86-2	Acetophenone	C_8H_8O	Ketones	6.33±0.50^{c}	5.20±0.47^{c}	10.09±0.31^{b}	14.76±0.36^{a}	15.87±1.11^{a}
18	1081	18409-17-1	(*E*)-2-Octen-1-ol	$C_8H_{16}O$	Alcohols	n.d.	n.d.	5.15±0.01^{a}	n.d.	2.33±0.12^{b}
19	1083	5989-33-3	(*Z*)-Linalool oxide (furanoid)	$C_{10}H_{18}O_2$	Alcohols	8.08±0.67^{c}	8.49±0.34^{c}	85.57±7.42^{a}	15.60±0.26^{b}	14.07±0.77^{bc}
20	1087	111-87-5	1-Octanol	$C_8H_{18}O$	Alcohols	n.d.	4.25±0.01	n.d.	n.d.	n.d.
21	1090	13360-65-1	3-Ethyl-2,5-dimethyl-pyrazine	$C_8H_{12}N_2$	Heterocyclics	0.57±0.04^{a}	n.d.	n.d.	n.d.	0.31±0.00^{b}
22	1098	34995-77-2	(*E*)-Linalool oxide (furanoid)	$C_{10}H_{18}O_2$	Alcohols	13.05±0.92^{c}	38.57±2.35^{b}	206.03±18.29^{a}	24.48±0.67^{bc}	20.14±0.93^{c}

23	1102	821-55-6	2-Nonanone	C9H18O	Ketones	n.d.	n.d.	n.d.	2.04±0.04	n.d.

Table S2. *Cont.*

No	RI[a]	CAS	Compounds	Formula	Category	Concentration (μg/L)[b]				
						GT	JT	OT	MT	RT
24	1104	38284-27-4	3,5-Octadien-2-one	C8H12O	Ketones	3.88±0.22^{a}	n.d.	3.54±0.01^{b}	n.d.	3.95±0.06^{a}
25	1105	93-58-3	Methyl benzoate	C8H8O2	Esters	n.d.	n.d.	n.d.	22.48±0.28	n.d.
26	1107	7299-41-4	*β*-Terpineol	C10H18O	Alcohols	3.27±0.30	n.d.	n.d.	n.d.	n.d.
27	1112	78-70-6	Linalool	C10H18O	Alcohols	57.82±3.49^{c}	5040.74±121.95^{a}	49.82±3.80^{c}	84.49±2.45^{b}	89.46±1.63^{b}
28	1114	124-19-6	Nonanal	C9H18O	Aldehydes	1.74±0.08^{c}	n.d.	7.85±0.77^{a}	7.68±0.68^{a}	4.40±0.26^{b}
29	1115	29957-43-5	Hoterienol	C10H16O	Alcohols	14.86±0.60^{a}	n.d.	8.87±0.03^{c}	15.31±0.44^{a}	12.18±0.58^{b}
30	1123	1632-73-1	Fenchol	C10H18O	Alcohols	n.d.	n.d.	1.13±0.01	n.d.	n.d.
31	1125	60-12-8	Phenylethyl Alcohol	C8H10O	Alcohols	20.84±0.91^{d}	23.26±1.35^{c}	21.59±0.56^{cd}	510.37±1.50^{a}	36.07±1.13^{b}
32	1138	673-84-7	2,6-Dimethyl-2,4,6-octatriene	C10H16	Hydrocarbons	n.d.	1.67±0.00	n.d.	n.d.	n.d.
33	1151	76-22-2	Camphor	C10H16O	Ketones	n.d.	n.d.	3.41±0.02	n.d.	n.d.
34	1153	89-79-2	Isopulegol	C10H18O	Alcohols	n.d.	n.d.	0.84±0.01	n.d.	n.d.
35	1158	53447-45-3	Lilac aldehyde A	C10H16O2	Aldehydes	n.d.	n.d.	1.18±0.01	n.d.	n.d.
36	1161	89-80-5	(±)-Menthone	C10H18O	Ketones	n.d.	n.d.	12.83±0.16	n.d.	n.d.
37	1171	491-07-6	Isomenthone	C10H18O	Ketones	n.d.	n.d.	4.37±0.09	n.d.	n.d.
38	1173	140-11-4	Benzyl acetate	C9H10O2	Esters	11.30±0.59^{c}	2382.41±3.28^{a}	17.46±0.30^{b}	23.49±1.07^{b}	21.43±0.84^{b}
39	1178	39028-58-5	(*E*)-Linalool oxide (pyranoid)	C10H18O2	Alcohols	2.98±0.29^{c}	n.d.	33.41±0.71^{a}	5.86±0.09^{b}	4.20±0.15^{bc}
40	1181	1490-04-6	Menthol	C10H20O	Alcohols	n.d.	n.d.	25.48±0.08^{a}	n.d.	1.63±0.05^{b}
41	1181	93-89-0	Ethyl benzoate	C9H10O2	Esters	n.d.	644.48±11.23	n.d.	n.d.	n.d.
42	1184	14009-71-3	(*Z*)-Linalool oxide (pyranoid)	C10H18O2	Alcohols	18.44±0.33^{e}	23.87±0.03^{d}	110.39±0.40^{a}	30.64±0.92^{b}	25.24±0.67^{c}
43	1195	16491-36-4	(*Z*)-3-Hexenyl butyrate	C10H18O2	Esters	1.68±0.04^{b}	16.44±0.09^{a}	2.25±0.08^{b}	2.74±0.23^{b}	1.88±0.12^{b}
44	1200	98-55-5	*α*-Terpineol	C10H18O	Alcohols	7.44±0.70^{e}	10.24±0.05^{c}	15.47±0.30^{a}	12.31±0.94^{b}	8.73±0.40^{d}

45	1203	119-36-8	Methyl salicylate	C8H8O3	Esters	13.91±0.51[c]	436.68±13.15[a]	8.91±0.55[d]	21.72±0.76[b]	20.12±0.65[b]
46	1213	112-31-2	Decanal	C10H20O	Aldehydes	0.76±0.02[c]	n.d.	1.36±0.03[b]	3.76±0.22[a]	1.59±0.12[b]
47	1222	4748-78-1	4-Ethyl-benzaldehyde	C9H10O	Aldehydes	24.52±0.57[e]	30.46±0.01[d]	34.98±0.15[c]	70.12±2.09[a]	59.56±1.90[b]

Table S2. *Cont.*

No	RI[a]	CAS	Compounds	Formula	Category	Concentration (μg/L)[b]				
						GT	JT	OT	MT	RT
48	1227	432-25-7	*β*-Cyclocitral	C10H16O	Aldehydes	2.64±0.09	n.d.	n.d.	n.d.	n.d.
49	1229	2601-13-0	2-Methylbutyl hexanoate	C11H22O2	Esters	n.d.	62.62±1.63[c]	n.d.	98.59±0.38[a]	88.22±0.95[b]
50	1237	106-25-2	Nerol	C10H18O	Alcohols	2.49±0.34[c]	3.71±0.02[b]	5.35±0.03[a]	3.90±0.31[b]	2.87±0.33[c]
51	1242	2270-60-2	Methyl citronellate	C11H20O2	Esters	n.d.	n.d.	0.78±0.01	n.d.	n.d.
52	1260	89-81-6	Piperitone	C10H16O	Ketones	n.d.	n.d.	5.18±0.08	n.d.	n.d.
53	1263	106-24-1	Geraniol	C10H18O	Alcohols	124.49±6.26[b]	129.97±1.27[b]	96.15±3.78[d]	161.98±2.84[a]	115.97±1.87[c]
54	1275	4179-19-5	3,5-Dimethoxytoluene	C9H12O2	Others	n.d.	n.d.	n.d.	4.53±0.16[b]	211.21±4.25[a]
55	1278	141-27-5	(*E*)-3,7-Dimethyl-2,6-octadienal	C10H16O	Aldehydes	n.d.	n.d.	2.21±0.06	n.d.	n.d.
56	1293	112-05-0	Nonanoic acid	C9H18O2	Acids	15.42±1.13[d]	24.87±0.51[c]	62.75±0.16[a]	32.60±0.14[b]	32.39±1.28[b]
57	1306	120-72-9	Indole	C8H7N	Heterocyclics	35.70±1.00[c]	1399.56±24.84[a]	24.55±0.21[d]	1029.78±63.32[b]	35.33±0.23[c]
58	1321	36431-72-8	Theaspirane	C13H22O	Heterocyclics	2.40±0.03[c]	n.d.	31.34±0.40[a]	n.d.	9.50±0.26[b]
59	1331	110-42-9	Decanoic acid methyl ester	C11H22O2	Esters	1.88±0.10[d]	5.50±0.02[b]	8.11±0.74[a]	4.41±0.32[c]	4.75±0.30[c]
60	1350	134-20-3	Methyl anthranilate	C8H9NO2	Esters	n.d.	59.41±0.12[a]	n.d.	6.02±0.04[b]	n.d.
61	1357	74367-31-0	2-Ethyl-3-hydroxyhexyl 2-methylpropanoate	C12H24O3	Esters	n.d.	13.29±0.02	n.d.	n.d.	n.d.
62	1365	97-53-0	Eugenol	C10H12O2	Phenols	n.d.	n.d.	3.32±0.10	n.d.	n.d.
63	1369	104-61-0	*γ*-Nonanolactone	C9H16O2	Heterocyclics	n.d.	n.d.	7.52±0.15	n.d.	n.d.
64	1372	4698-08-2	Geranic acid	C10H16O2	Acids	42.09±0.42[b]	27.77±0.22[c]	n.d.	48.35±0.21[a]	48.55±1.35[a]
65	1386	334-48-5	n-Decanoic acid	C10H20O2	Acids	6.28±0.44[d]	14.66±0.11[c]	n.d.	20.81±0.36[a]	19.27±0.21[b]
66	1406	488-10-8	Jasmone	C11H16O	Ketones	13.21±0.39[d]	16.03±0.03[b]	13.46±0.16[d]	21.90±0.39[a]	14.54±1.14[c]

67	1415	93-15-2	Methyleugenol	C11H14O2	Others	n.d.	n.d.	3.54±0.14	n.d.	n.d.
68	1432	17283-81-7	Dihydro-*β*-ionone	C13H22O	Ketones	n.d.	n.d.	10.78±0.02^{a}	n.d.	1.87±0.13^{b}
69	1443	127-41-3	*α*-Ionone	C13H20O	Ketones	1.14±0.03^{c}	1.00±0.00^{c}	21.06±0.03^{a}	5.57±0.18^{b}	1.27±0.10^{c}
70	1454	91-64-5	Coumarin	C9H6O2	Heterocyclics	16.81±0.49^{c}	15.58±0.22^{c}	n.d.	36.20±0.63^{a}	27.37±1.44^{b}

Table S2. *Cont.*

No	RI[a]	CAS	Compounds	Formula	Category	Concentration (μg/L)[b]				
						**GT**	**JT**	**OT**	**MT**	**RT**
71	1457	103-52-6	*β*-Phenylethyl butyrate	C12H16O2	Esters	n.d.	n.d.	n.d.	26.68±0.49	n.d.
72	1462	3293-47-8	Dihydro-*β*-ionol	C13H24O	Alcohols	n.d.	n.d.	28.26±0.09	n.d.	n.d.
73	1468	3796-70-1	Geranylacetone	C13H22O	Ketones	2.68±0.07^{e}	4.62±0.11^{d}	11.52±0.29^{a}	8.30±0.51^{b}	5.35±0.13^{c}
74	1482	706-14-9	*γ*-Decalactone	C10H18O2	Heterocyclics	n.d.	n.d.	663.09±2.47	n.d.	n.d.
75	1486	112-53-8	1-Dodecanol	C12H26O	Alcohols	3.11±0.33^{d}	5.49±0.01^{c}	11.88±0.04^{a}	7.38±0.32^{b}	5.69±0.41^{c}
76	1495	79-77-6	(*E*)-*β*-Ionone	C13H20O	Ketones	n.d.	n.d.	330.89±2.78	n.d.	n.d.
77	1500	705-86-2	*δ*-Decalactone	C10H18O2	Heterocyclics	n.d.	11.88±0.02	n.d.	n.d.	n.d.
78	1533	483-76-1	*δ*-Cadinene	C15H24	Hydrocarbons	0.74±0.02^{b}	1.18±0.00^{a}	n.d.	n.d.	n.d.
79	1540	17092-92-1	Dihydroactinidiolide	C11H16O2	Heterocyclics	26.96±0.71^{e}	35.21±0.39^{d}	52.94±0.40^{a}	43.87±0.70^{b}	42.15±0.81^{c}
80	1580	25152-85-6	(*E*)-3-Hexenyl benzoate	C13H16O2	Esters	n.d.	32.18±0.03	n.d.	n.d.	n.d.
81	1581	143-07-7	Dodecanoic acid	C12H24O2	Acids	11.61±3.08^{d}	35.56±0.03^{b}	38.84±1.25^{b}	61.47±3.54^{a}	22.35±1.92^{c}
82	1585	198991-79-6	Germacrene-*δ*-4-ol	C15H26O	Alcohols	n.d.	6.88±0.01	n.d.	n.d.	n.d.
83	1612	77-53-2	Cedrol	C15H26O	Alcohols	3.07±0.20^{d}	4.34±0.01^{b}	4.64±0.04^{a}	4.57±0.10^{a}	3.73±0.11^{c}
84	1616	1139-30-6	Caryophyllene oxide	C15H24O	Others	n.d.	n.d.	4.48±0.05	n.d.	n.d.
85	1619	28305-60-4	*β*-Oplopenone	C15H24O	Ketones	n.d.	3.66±0.18	n.d.	n.d.	n.d.
86	1626	102488-09-5	3-Hydroxy-*β*-damascone	C13H20O2	Ketones	1.11±0.00	n.d.	n.d.	1.65±0.23	n.d.
87	1654	19912-62-0	*τ*-Muurolol	C15H26O	Alcohols	2.23±0.01^{d}	7.75±0.03^{a}	3.63±0.07^{b}	n.d.	2.41±0.03^{c}
88	1658	1211-29-6	Methyl jasmonate	C13H20O3	Esters	14.94±0.35^{b}	9.18±0.14^{c}	5.41±0.13^{c}	61.43±4.68^{a}	8.78±0.58^{c}

89	1667	481-34-5	*α*-Cadinol	C15H26O	Alcohols	3.31±0.19[b]	12.76±0.35[a]	n.d.	n.d.	n.d.
90	1683	2306-78-7	Nerolidyl acetate	C17H28O2	Esters	n.d.	n.d.	n.d.	14.75±0.85	n.d.
91	1753	1911-78-0	Oplopanone	C15H26O2	Ketones	n.d.	36.57±0.04	n.d.	n.d.	n.d.
92	1769	544-63-8	Tetradecanoic acid	C14H28O2	Acids	4.83±0.21[d]	6.44±0.12[c]	7.34±0.09[b]	9.30±0.38[a]	n.d.
93	1797	638-36-8	Phytane	C20H42	Hydrocarbons	n.d.	n.d.	1.89±0.03	n.d.	n.d.
94	1944	373-49-9	Palmitoleic acid	C16H30O2	Acids	4.96±0.25[b]	7.74±0.13[a]	7.69±0.22[a]	n.d.	7.65±0.05[a]

Table S2. *Cont.*

No	RI[a]	CAS	Compounds	Formula	Category	Concentration (μg/L)[b]				
						GT	JT	OT	MT	RT
95	1963	57-10-3	n-Hexadecanoic acid	C16H32O2	Acids	16.46±0.03[e]	34.56±0.05[b]	28.23±0.07[c]	42.00±2.58[a]	25.49±0.70[d]
96	1993	3452-07-1	1-Eicosene	C20H40	Hydrocarbons	n.d.	1.50±0.00	n.d.	n.d.	n.d.
97	2136	112-80-1	Oleic Acid	C18H34O2	Acids	4.90±0.26[c]	4.71±0.13[c]	5.93±0.20[b]	8.43±0.28[a]	n.d.
98	2138	463-40-1	*α*-Linolenic acid	C18H30O2	Acids	n.d.	n.d.	6.68±0.60	n.d.	n.d.
99	2155	57-11-4	Octadecanoic acid	C18H36O2	Acids	3.60±0.09[e]	6.05±0.23[c]	4.79±0.29[d]	10.21±0.14[a]	6.58±0.28[b]
100	2166	1599-67-3	1-Docosene	C22H44	Hydrocarbons	2.05±0.05[b]	2.81±0.11[a]	n.d.	n.d.	n.d.
101	2178	629-54-9	Hexadecanamide	C16H33NO	Others	4.12±0.71[a]	3.24±0.01[a]	3.87±0.28[a]	n.d.	n.d.

Note: [a] Retention index (RI) by HP-5MS capillary chromatography column. [b] Green tea (GT), Jasmine tea (JT), Osmanthus tea (OT), Michelia tea (MT) and Rose tea (RT). n.d., the compounds were not detected. Values in data with different letters (a–e) in the same row are significantly different ($p < 0.05$)
